# Chip-Based Optoacoustic Single-Cell Detection in Flow Using Point-Source Optimized Surface Acoustic Wave Transducers

**DOI:** 10.1021/acsami.4c20182

**Published:** 2025-02-25

**Authors:** Simon Göllner, Melanie Colditz, Yishu Huang, Hagen Schmidt, Andreas Winkler, Andre C. Stiel

**Affiliations:** † Institute of Biological and Medical Imaging, Helmholtz Zentrum München, Neuherberg, Bavaria 85764, Germany; ‡ Leibniz Institute for Solid State and Materials Research, SAWLab Saxony, Dresden, Saxony 01069, Germany; § Protein Engineering for Superresolution Microscopy Lab, University of Regensburg, Regensburg, Bavaria 93053, Germany; ⊥ Chair of Biological Imaging, Central Institute for Translational Cancer Research (TranslaTUM), School of Medicine and Health & School of Computation, Information and Technology, Technical University of Munich, Munich 80333, Germany

**Keywords:** single cell measurements, microfluidics, opto-/photoacoustic, surface acoustic waves, interdigital transducers

## Abstract

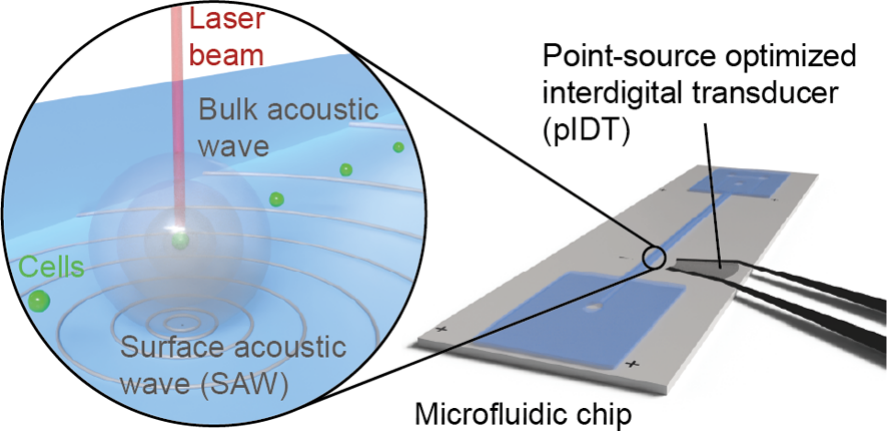

Sensitive measurement of the optoacoustic (OA, also photoacoustic) properties of cells in flow is highly desirable, as it provides information about the optical absorption properties of cellular compounds. Hence, optoacoustic spectral characteristics can deliver information about the cell state or disease parameters, but can also be used for high-throughput cell sorting by intrinsic properties without additional fluorescence labeling. The current implementation of optoacoustic measurements of cells in a microfluidic context typically relies on piezoelectric (ultrasound) transducers attached to the microfluidic chip, whereby the transducer records the ultrasound signal originating from absorbing species in cells when excited by laser pulses. The arrangement of the transducer outside of the microfluidic chip leads to the challenge of signal integration over a larger area and coupling interlayer effects resulting in attenuation and a reduction of sensitivity. Moreover, the placement of the bulky transducer outside of the chip prevents the exploitation of the full advantages of microfluidics. As a solution, we demonstrate the use of point-source optimized interdigital transducers (pIDTs) directly fabricated on the surface of the microfluidic chip for the detection of surface acoustic waves (SAW) from single cells in continuous flow. The SAW is excited by bulk acoustic waves originating from the optoacoustic effect of absorbing species inside the cells illuminated by laser light. The use of these highly focused pIDTs and on-chip lithographically fabricated hard-wall microchannels allows the detection of SAW with a spatial resolution on the order of the cell diameter directly on-chip, offering the possibility of miniaturization, parallelization, and cheap mass production.

## Introduction

**Figure 1:**
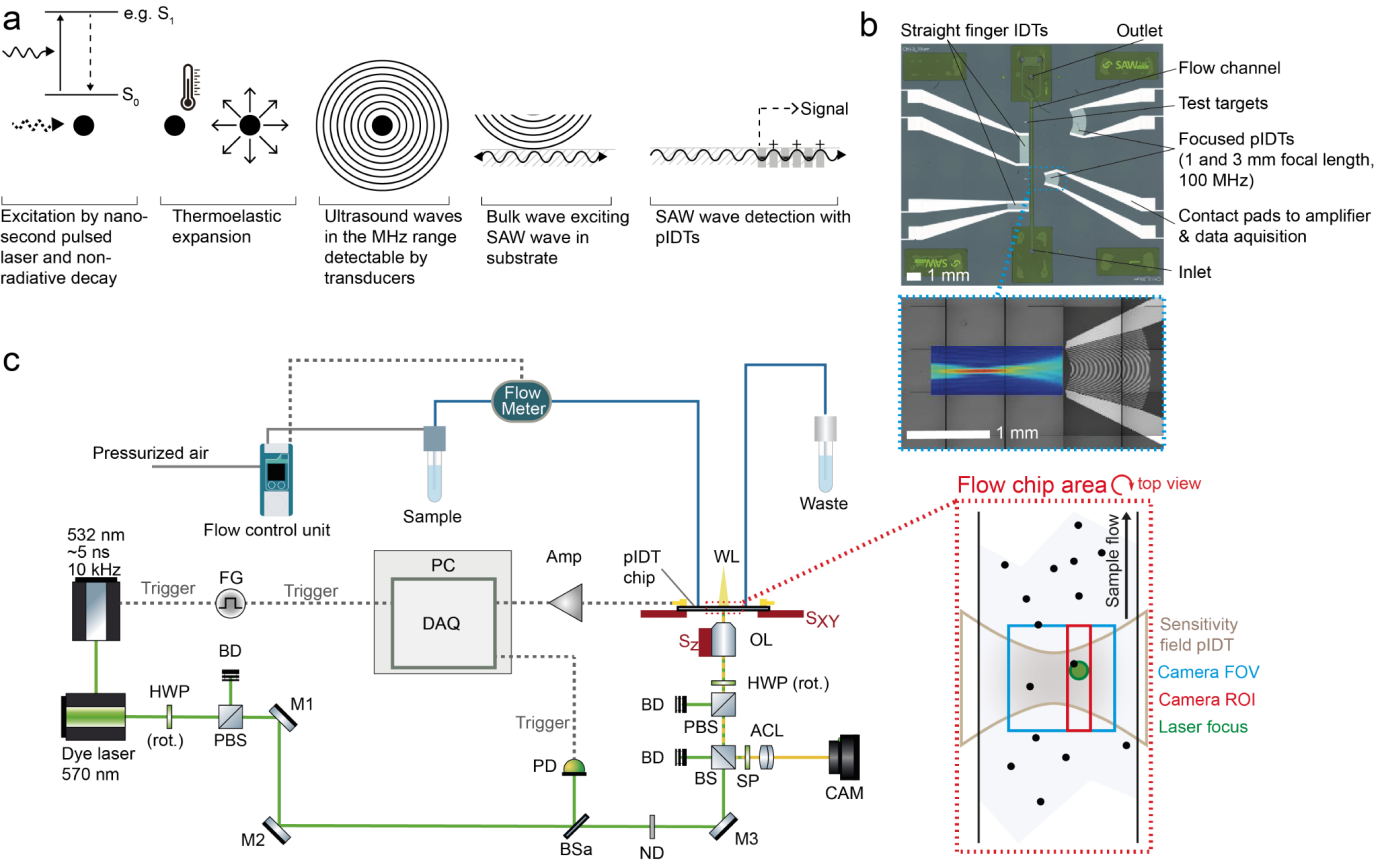
Optoacoustic principle and experimental setup. (a) Schematic of the five main steps of optoacoustic signal generation and detection. (b) Exemplary chip layout with 4 different IDTs (two with straight and two with focused electrode design). The inset shows the sensitive area of a 100 MHz pIDT (1 mm focal length) in terms of the lateral surface-normal amplitude distribution measured by laser-Doppler vibrometry. Please note that the field is shown on a chip without the attached channel. (c) Optical and microfluidic setup for pulsed laser excitation, OA detection, and white-light imaging of cells in flow. ACL: achromatic lens, Amp: variable gain amplifier, BD: beam dump, BSa: 90/10 beam splitter, BS: 50/50 beam splitter, CAM: camera, DAQ: data acquisition card, FG: function generator, HWP: half-wave plate, M1–M3: dielectric mirror, ND: neutral density filter wheel, OL: objective lens, PBS: polarizing beam splitter, PD: photodiode, SP: short-pass filter, S_XY_: xy-translation stage, S_Z_: z-translation stage, and WL: white light. Inset: Schematic top view of the microfluidic channel showing the alignment of the acquired camera frame, the camera ROI used to coregister the OA signal with the camera, the laser focus, and the sensitivity field of the pIDT.

The photoacoustic (also called optoacoustic, OA) effect is a phenomenon that was first studied by Bell in 1880.^1^ The effect describes the absorption of a photon by a suitable substance and the dissemination of the energy by nonradiative pathways, leading to rapid heating and thermoelastic expansion. Such substances can be natural intrinsic absorbers, such as hemoglobin or melanin, or exogenous contrast agents, such as synthetic dyes, nanoparticles, or chromophore-bearing proteins. If the transient illumination occurs on a very short time scale, the change in local pressure due to the thermoelastic expansion yields measurable ultrasound signals with frequencies in the (radio frequency) MHz range^2^ (Figure 1a).

Depending on how efficiently the absorbed energy can be transformed into an acoustic wave (i.e., the optoacoustic quantum yield, or in other words, *η*_th_ in the description of the initial photoacoustic pressure *p*_0_ = Γ*η*_th_*μ*_a_*F*, with Γ being the Grüneisen parameter, *μ*_a_ the optical absorbance, and *F* the fluence), the spectral signature of a chromophore OA signal strongly resembles its absorbance spectrum.^3^ Unlike absorption spectroscopy, which quantifies the remaining light after passing through a sample, OA detection measures the absorbed energy, which is subsequently “reemitted” as an acoustic signal. This can lead to a lower influence of the background on the measurement^4^ and could therefore be an advantage for the OA measurement in contrast to the absorption measurement when measuring very small amounts of absorbing material—such as in a single cell.

Absorbance measurements from single cells in a microfluidic high-throughput fashion are highly challenging due to the small amount of absorbing material in the beam path and the short transit time available for the measurement (e.g., assuming that the beam has the diameter of a red blood cell, one could expect an OD_570_ of approximately 0.135, with millisecond transit times typical for the used microfluidic layouts). Such measurements are possible by tightly focusing the beam inside the chip, requiring additional optics or high-speed cameras,^5−8^ using fiber optics directly in the channel material^9^ or using optical resonator technology. The latter allows multiple passages of the beam through the cell to achieve a sufficient change in accumulated absorbance^10^ and improves the signal-to-noise ratio. This makes such technologies rather complex, costly, and not easily parallelizable.

Measuring the absorptive properties of a cell is highly desirable because many biochemical components within a cell absorb light, unlike the relatively few that fluoresce. However, fluorescence remains the primary modality in fluidic cytometry devices such as fluorescence-activated cell sorting (FACS). Hence, OA measurements, which enable the detection of numerous absorbing but nonfluorescent cellular compounds, might pose a promising route to capture more information from a cell without the need for additional labeling. Moreover, the OA signal can be measured orthogonal to fluorescence, meaning the incorporation of an inline OA detection into any fluorescence detection device would allow crosstalk-free detection of both characteristics. So far, OA measurements in fluidic systems have been almost entirely demonstrated by piezoelectric ultrasound transducers, i.e., employing so-called bulk acoustic waves (BAW).^11,12^

Examples of OA measurements based on piezoelectric transducers show the detection of circulating melanoma or labeled tumor cells,^13−16^ as well as red blood cells and white blood cells, made possible by additional ultrasound^17,18^ or labeling.^19^ It has been demonstrated both in vitro and in vivo (mouse ear capillary). Especially for in vitro use, the – from an acoustic and microtechnological point of view – complex experimental setups hinder the exploitation of the high miniaturization, parallelization, and general economic advantages typically associated with microfluidics technologies. Beyond that, a major obstacle for achieving high sensitivity and high signal-to-noise ratio (SNR) is the behavior of ultrasonic BAW emitted from cells in the flow channel. These wave modes encounter multiple material transitions with varying acoustic impedances, reflections, refractions, and possible mode conversions, which particularly attenuate high-frequency signal components.^20^ Nonetheless, few optoacoustic BAW cell-detection approaches have been demonstrated successfully, realizing single-cell detection of bacteria expressing contrast agents,^21^ melanoma cells, or carbon nanoparticles.^22^

Here, we suggest an alternative approach employing the detection of surface acoustic waves (SAW) excited by the BAW pulse originating from a cell or particle when interacting with the fluid–solid interface, i.e., the chip surface. The underlying effect is the reverse of the effect commonly used in SAW-driven acoustofluidics actuation.^23,24^ For an angle of incidence of the BAW on the substrate that corresponds to the so-called Rayleigh angle, which is determined by the phase velocities of BAW and SAW, the phase matching condition for an optimal BAW-to-SAW conversion is fulfilled despite the strongly different acoustic impedances of liquid and crystalline substrate. In our setup, the liquid BAW emanating from the laser-excited microobject in the channel hits the substrate surface in the focal area of the pIDTs at the Rayleigh angle and is thereby converted into a phase-matched SAW without any shift in frequency. This locally excited SAW spectrum propagates along the substrate surface with a wavefield determined by the shape of the acoustic slowness curve and with a penetration depth into the substrate of only about one wavelength. Therefore, the wave energy is confined near the crystal surface, while the SAW passes under the hard channel wall and experiences some partial reflection. Outside the channel, the SAW propagates along the free crystal surface with virtually no attenuation and is received by the focused pIDTs, which have been patterned with high geometric resolution on the surface of the piezoelectric substrate by using planar techniques. Due to their adapted electrode geometry, the SAW spectrum is converted by the pIDTs with high efficiency into the electrical signal that is finally fed to the measuring electronics. Being indispensable to modern telecommunication devices, SAW technology has elaborated a rich toolbox of IDT topologies to meet very different requirements in high-frequency filtering, which also addresses special needs of SAW sensors as the second generation of SAW applications like used here. Currently, the only demonstrations of detection of OA-generated ultrasonic waves from cells using IDTs are based on (unfocused) IDTs with straight electrodes and an amassment of cells in the focal volume in a stationary setup without liquid flow.^25,26^ However, the sensitivity of straight IDTs for point-excited SAW is low, hindering so far high SNR single-cell measurement in flow.

In this work, we demonstrate the successful detection of beads and single cells (red blood cells (RBCs) and B16 melanoma cells) in continuous flow with a high SNR. For this, we utilized microacoustofluidic chips combining on-chip, hard-walled microfluidic channels and point-source optimized IDTs (pIDTs) on a planar piezoelectric substrate. The chip consists of the piezoelectric single-crystal substrate with both the flow channel and the pIDTs on top of its surface and is manufactured on the wafer scale. This combination allows good capability for highly reproducible, cost-efficient mass production of OA cell detection chips and a future transfer to small real-world OA analysis devices. Moreover, the high-resolution photolithographic manufacturing process enables precise alignment of the microfluidic channel, the pIDT’s focus region, and laser-illuminated spatial focus region within the well-defined channel geometry, ensuring efficient signal conversion over the whole signal path.

## Results

In total, we tested seven point-focused IDT (pIDT) configurations using a custom-built setup (Figure 1c) and an analysis routine (Figure S1), with an exemplary image of a single microfluidic chip shown in Figure 1b. The tested pIDTs comprised four layouts with a focal length of 1 mm and were designed for center frequencies of 10, 25, 50, and 100 MHz, respectively, supplemented by three layouts with a focal length of 3 mm designed for 25, 50, and 100 MHz, respectively. A 3 mm focal length 10 MHz IDT with the appropriate number of finger pairs would have been too large for the given chip holder. As an optical control, we monitored the flow path with a camera to confirm events, i.e., cell transitions, detected via the pIDTs. The set flow rate of 0.5 μL/min (beads and RBCs) or 1 μL/min (B16 cells) results in 2 to 3 camera frames per cell or particle. To identify objects passing through the interrogation volume (the region of interest, ROI), we calculated a baseline for each pixel and averaged the absolute deviation from the baselines for the whole ROI. The noisy camera images make unequivocal detection of passing objects challenging; hence, we used the coregistration success (hit rate) with respect to the event thresholding of the camera signal (Figures S1 and S2 and Table S1) with a conservative threshold (5× standard deviation of noise) as a true positive criterion. To evaluate this threshold, the receiver operating characteristics (ROC) were calculated and are shown exemplarily for the 50 MHz, 3 mm focal length pIDT (Figure S3). This pIDT was chosen for this since it performed well in the detection of all three particle types. The area under the curve (AUC) values for B16, RBC, and beads were 0.975, 0.977, and 0.973, respectively (Figure S3), indicating a high level of discrimination between true and false positives across all particle types.

**Figure 2:**
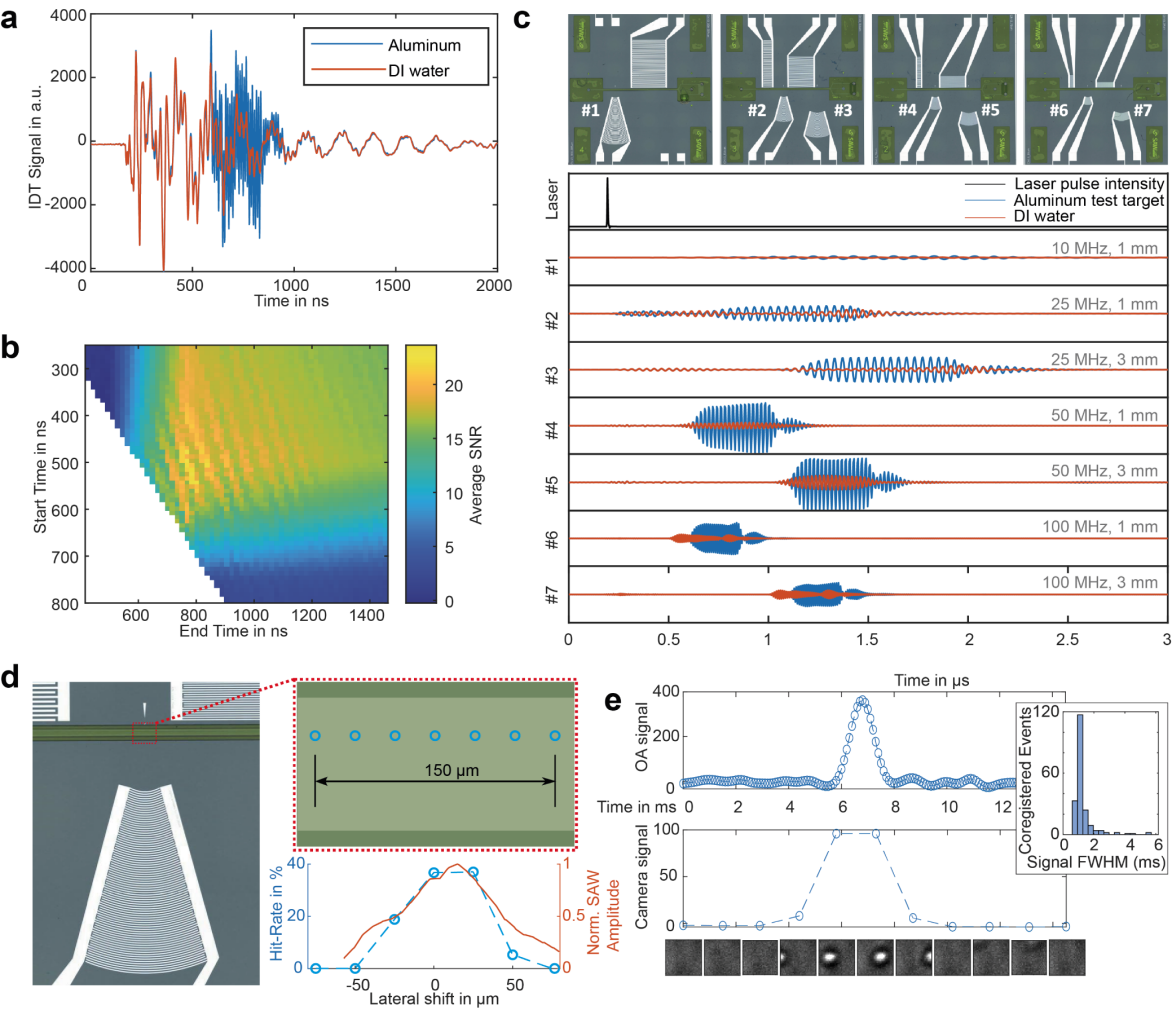
Characterization of the pIDTs and optimization of the OA signal processing (a) Exemplary pIDT signal traces received from focusing the laser at the microfluidic channel containing only buffer and at the aluminum test target, respectively. (b) Time-gating optimization of the pIDT signal. Averaged SNR of coregistered bead transits for the 100 MHz pIDT shows an optimal time-gating to detect the bead OA signal from 550 to 800 ns. To evaluate the signal acquired from pIDT, we calculated the FFT of the signals for different time-gating settings. As a metric, we chose the SNR of the coregistered events (see Material and Methods for details), where the OA signal height of the event is divided by the noise (standard deviation of ∼1000 trigger events with no visible particle transition). For each pIDT, an optimal start and end point with the highest SNR can be found. The choice of time-gating settings depends on the physical layout of the pIDT (number of finger pairs, focal length, and distance to the microfluidic channel). (c) Photographs of tested chips and OA response to pulsed illumination of aluminum test targets measured by the respective pIDTs (bandpass filtered ±10% of the center frequency, note response in Figure 1c is not bandpass filtered). First plot shows the laser pulse detected by the photodiode, the following pIDTs of indicated center frequency, and focal length. (d) Evaluating the lateral sensitivity of the pIDT. Left: representative photograph of a pIDT. Top-right: enlarged schematic illustrating the laser focus points of a raster scan, marked as blue circles. Bottom-right: sensitivity evaluation performed by conducting 10 s measurements at each focus point using beads. The hit rates from each measurement are displayed alongside the surface-normal SAW amplitude profile obtained through laser Doppler vibrometry. (e) Full width at half-maximum (fwhm) analysis of beads in flow. Top: time trace of the optoacoustic (OA) signal showing the transition of a single polystyrene bead. Center: histogram depicting the distribution of OA signal widths during the transition of 3 μm diameter beads over a 10 s measurement period. Bottom: representative camera frames captured at specific time points during the bead transition. Inset: histogram illustrating the signal width distribution.

For an initial characterization of the frequency response of the pIDTs, we illuminated an aluminum test target, i.e., a small metal patch next to the channel produced together with the IDTs during lithography. The obtained travel time and frequency responses of the SAW pulse match the theoretical characteristics (Figures 2a,c and S4). Toward exploring the general system characteristics, we screened 3 μm blackened polystyrene beads in deionized water (Figure S5). The travel time of the SAW from the object to the pIDT was controlled by measuring the SNR for multiple possible selection windows (Figure 2a,b). To characterize the lateral sensitivity field of pIDTs, we show exemplarily for one pIDT the hit rate of multiple measurements of beads at different lateral positions in the microfluidic channel (Figure 2c), yielding a sensitivity field of ∼50 μm (fwhm), matching the expected fwhm value obtained by laser-optic wave field characterization (Figure 2c). The coregistration with the camera allowed us to correlate detected OA signals from the pIDTs with camera recordings. The averaged width of the OA signals detected for beads in transit matched theoretical expectations based on the focus size, laser pulse-repetition rate, and flow speed (Figure 2d and the Methods section).

**Figure 3:**
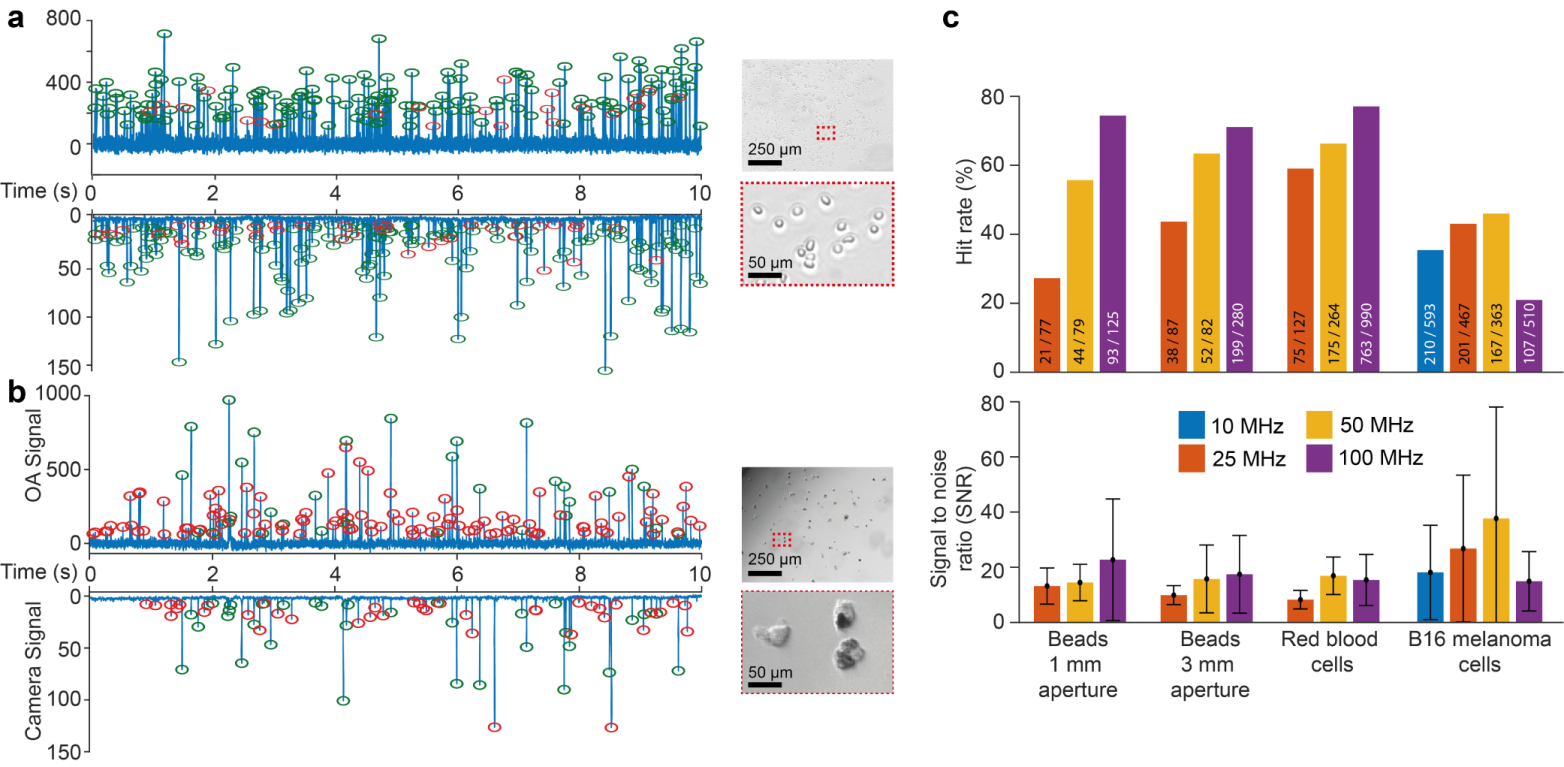
Exemplary traces of red blood cells and B16 melanoma cells and pIDT signal characteristics for each sample. (a) Trace of red blood cells. Green circles indicate coregistered signals and red circles indicate unassigned in the respective other trace. This measurement was performed using the 100 MHz pIDT with a 3 mm focal length. Right: microscope image of an aliquot of red blood cells (scale bar, 125 μm) and a section of it (scale bar, 25 μm). (b) Trace of B16 melanoma cells. Green circles indicate coregistered signals and red unassigned in the respective other trace. This measurement was performed using the 50 MHz pIDT with 3 mm focal length. 40 events were coregistered out of 75 events detected by the camera (53% hit rate). Right: microscope image of an aliquot of B16 melanoma cells (scale bar = 250 μm) and a section of it (scale bar = 50 μm). (c) Characteristics of the measurements regarding the OA signal, SNR, and hit rate. Bars depict the mean; error bars depict the standard deviation. Due to the dimension of the pIDT, there is no 10 MHz chip with a 3 mm focal length. Red blood cells could not be detected using a 10 MHz pIDT.

Next, we tested the detection of two medically relevant cell types, namely, red blood cells (diameter ∼7 μm, Figure 3a) and B16 melanoma cells (∼23 μm, Figure 3b). For the two cell types with clearly different diameters, we observed significant differences in the OA signals and hit rates depending on the chosen pIDT (Figures 3c and S6). This is despite the laser focus being smaller than both cell types and the distribution of absorbing materials in the B16 cells being highly inhomogeneous (see the inset in Figure 3b). For RBCs, 50 and 100 MHz pIDTs show a significantly higher SNR (*p* < 0.001) than the 25 MHz pIDT (SNR 8 for 25 MHz; SNR 17 for 50 MHz; SNR 20 for 100 MHz; Table S1). The 100 MHz pIDT has the highest hit rate for RBCs with 78%. In contrast, the 25 and 50 MHz pIDTs show the highest SNR for B16 melanoma cells (*p* < 0.001). There is no significant difference between the 25 and 50 MHz pIDTs when measuring B16 melanoma cells regarding the SNR and hit rate. Together with the pIDT frequency-dependent results from the beads, this highlights the frequency selectiveness due to the relatively narrow bandwidth of pIDTs (about 13.3%, 10%, 10%, and 8% of the respective center frequency of 10, 25, 50, and 100 MHz; Figure S4c). This affects the signal strength, noise, signal-to-noise ratio (SNR), and eventual hit rate. For both focal lengths, the 100 MHz pIDT has a significantly higher SNR when detecting beads compared to 10 and 25 MHz (*p* < 0.05). Despite no significant difference in the SNR, there seems to be a trend regarding the hit rate when detecting beads: higher frequency causes a higher hit rate due to the more suitable frequency spectrum of the pIDT and the OA signal frequency as a function of object size.

Likely, this difference would be even stronger with a larger illumination area and focused flow. In this case, the OA signal frequency would be entirely dependent on object size and not a convolution of object size, illuminated area, and how an object was hit (fully or only partially illuminated). Due to this and the camera detection not matching 1:1 with the illuminated area (Figure 1c, inset), an ideal correlation between the OA signal measured by the pIDT and the camera image cannot be made (Figure S7). However, both cell types, red blood cells and melanoma cells, could be readily detected with hit rates of 77% and 46% for the best suitable IDTs.

For pIDTs with different focal lengths and frequencies, we obtained significantly different percentages of coregistration (hit rate), suggesting different detection sensitivities (Figure 3c and Table S1). However, a direct comparison of the OA signal intensities does not show a similarly clear trend (Figure S6). Likely, this is due to the obtained OA signal being correlated with the pIDT frequency optimum and conversion efficiency, object size, and laser focus. Similarly, the SNR follows a trend of object size/frequency but not as clearly as the hit rate. Likely, the lack of very clear numerical correlations is also associated with the detection of cells and beads not always being complete and uniform. Since the camera signal is averaged over the whole camera frame ROI, a small particle with high absorptivity can result in the same camera signal as a large cell with low absorptivity. To reduce the camera ROI, a higher frame rate and a higher resolution would be necessary. Furthermore, particles can be hit by the laser beam fully or (usually) partially, resulting in different OA signal strengths, and the position of optoacoustic interaction on the particle and in relation to the spatial position in the channel may also affect the propagation of the excited bulk acoustic wave and the subsequent conversion into a surface acoustic wave. For 3 μm diameter beads, we did not measure a significant OA signal difference between using focal lengths of 1 and 3 mm for the same center frequency (Figure 3c), indicating the low attenuation of the propagating SAWs.

## Conclusion

Here, we propose a new method for optoacoustic particle and cell detection incorporating pulsed laser excitation in hard-walled microfluidic channels – fabricated via wafer-scale lithography – with SAW detection by point-source optimized (i.e., point-focused) IDTs. Using this method, we demonstrate the sensitive detection of individual PS beads, mammalian red blood cells, and B16 melanoma cells in microfluidic flow, for the first time using SAW-based OA detection. This shows the feasibility of reading out absorption signatures of single cells in flow by using OA signals detected via SAW and pIDTs fully integrated into microfluidic chips. The integration allows for a much more compact build of a potential device than that possible with BAW transducers placed outside of the chip. This might open up several biomedical applications. However, for technology transfer, a range of aspects need to be addressed, foremost an improvement of sensitivity. Our limit of sensitivity (based on the two measured cell types) could be estimated to be an optical density of 0.05 at 570 nm for a single cell, yielding an SNR of 5. This is a factor of 2–3 below the sensitivity of works showing single-cell in flow detection with BAW transducers,^13,17,21,22^ although a comparison is challenging due to the lack of benchmark data. Similarly, purely optical methods (cameras, microspectrometers, or optical cavity-based approaches, e.g.^5,7,8,10^ seem to show a similar SNR; however, in the case of cameras, or scatter, they provide additional information content, e.g., cell shape and fine structure. Experimentally, our measurement could benefit from an improved placement of the cells within the ROI, e.g., due to a reduction of the channel height, or particle positioning, e.g., by a sheath flow. However, this optimization is quite complex, and besides the manufacturing accuracy, especially the altered liquid flow rate and cell/particle velocity must be considered. An increase in the particle speed, when reducing the channel height at a constant sample flow rate or by adding a sheath flow, reduces the particle time in the ROI. A reduction of the sample flow rate, however, can increase the influence of (bio)particle sedimentation, agglomeration, channel wall attachment, and clogging. On the side of the pIDTs, we see room for improvement when using design arrangements tailored to the micro-objects to be detected, in which the frequency and the focal length of the pIDTs as well as the channel height and width are directly tailored to the size and the expected OA response.

In line with this, our work identifies a correspondence between the pIDT center frequency, the size of the object, and its detectability based on the OA signal, as confirmed through the coregistration with the camera. This correlation is likely governed by how well the emitted US frequencies, depending on the object size, match the frequency of the pIDT. The operating frequency of the pIDTs can be easily controlled by adjusting the finger period, enabling the tailored fabrication of acoustofluidic chips for specific cell types. Additionally, pIDTs designed for multiple analysis frequencies can be arranged either downstream or in a radial configuration around a single measurement point, allowing for more versatile detection. Also, size measurements are theoretically possible via the ultrasound frequency being dependent on the emitting object. Beyond that, with the increasing popularity of OA imaging in the biomedical realm,^27,28^ such devices can accelerate the benchmarking and screening of genetically encoded contrast agents for OA imaging,^29^ particularly in directed evolution approaches. Major advantages of the detection of OA signals by pIDTs are the compact footprint of the setup, its high reproducibility and mass-producibility, and low unit costs, suggesting straightforward miniaturization and parallelization of the devices. In line with this, the effective excitation light fluences in the focus were approximately 130 μJ/mm^2^ (400 μJ/pulse at 10 kHz), which is in accordance with energies achieved by pulsed laser diodes (190 μJ/pulse@200 kHz).^30,31^ The latter are much cheaper and have a footprint smaller than the Q-switched laser sources used for this demonstration work. This allows envisioning small cost-effective devices to read out absorptive properties of cells, for example, for hematological applications related to hemoglobin content indicative of numerous pathophysiologies.^32^ Furthermore, the tailorable sensitivity field can also allow for laser diode-based multiwavelength measurements to determine blood oxygenation^33^ along a very short interrogation zone. SAWs can also enable other microfluidic functions relevant to such an OA system, including cell separation by size or density, fluid mixing at low Reynolds numbers, on-demand cell sorting, and cell focusing in the ROI. This capability makes compact, all-in-one devices possible, enabling an analysis without external sample preparation. Combining SAW actuators with additional detection methods, e.g., optical or electrical, could even enable a technology platform for micro total analysis systems (μTAS). This approach could enable multistage processing of complex biological fluids, starting with cell separation via SAW actuators and followed by specialized analytical methods. Lastly, SAW sensors can be applied to a variety of sensing tasks in the engineering and medical fields. In such sensors, a surface acoustic wave of known properties or excitation and transmission characteristics is typically altered by a (biological) medium present in the acoustic path.^34−37^ These changes can be analyzed and give insights into the altered mechanical and electrical properties of the acoustically excited material. Additionally, both light-acoustic and purely acoustic sensing properties can potentially be combined. In summary, we demonstrate that the absorptive properties of cells can be recorded in a microfluidic flow with high sensitivity using acoustically fully integrated chip devices. This approach enables effective miniaturization and parallelization, making it promising for potential (bio)medical applications.

## Material and Methods

### Acoustofluidic Chips and Periphery

The developed acoustofluidic chip was realized on single-crystal lithium niobate (black 128° YX-LiNbO_3_ with main SAW propagation along the *X*-direction, i.e., Euler angles (0°, 37.86°, 0°)) as a substrate for piezoelectric SAW excitation. The chips are double-side polished to allow undisturbed laser access to the channel in transmission from the rear side. They have lateral dimensions of 17.7 mm × 19 mm (width × length) at a thickness of 525 μm and carry all interdigital transducer electrodes, the microfluidic channel with dedicated OA interaction regions, as well as fluid connectors for inlet and outlet on the upper surface. Chip design was carried out using QCAD (Ribbonsoft GmbH).

Point-source optimized IDTs (pIDTs, Figures 1b and S3a) were prepared on the chip surface with center frequencies of 10, 25, 50, and 100 MHz and focal lengths of 1 and 3 mm, respectively. Due to size constraints on the chip, a 10 MHz pIDT with a 3 mm focal length could not be manufactured. Conventional IDTs with straight fingers were also tested; however, they did not yield sufficient signal intensity and were dismissed in preliminary tests. To adapt the IDTs to the OA case of point-shaped acoustic sources, they were designed as pIDTs with curved finger electrodes with a focal point located underneath the channel. Additionally, the IDTs are of the so-called split (or λ/8) finger type, instead of the solid (or λ/4) finger type, to reduce IDT internal reflections and thereby prevent undesired prolongation of received signal pulses. Several layouts of focused split-finger pIDTs have been realized with curvatures based on the specific anisotropic SAW excitation and propagation properties (e.g., angular dispersion of velocity and beam steering) of the substrate^38^ in combination with the mechanical properties of the IDT metallization. The overall angular aperture was 30° for all IDTs set symmetrically around the *X*-direction (i.e., *X* ± 15°). All pIDTs were fabricated by conventional patterning technology comprising photoresist structuring by rapid-prototyping laser photolithography (MLA 100, Heidelberg Instruments Mikrotechnik GmbH), e-beam deposition of electrode metallization (295 nm Al on 5 nm Ti, Clustertool Creamet 350 Cl 6, Creavac GmbH), and a subsequent liftoff procedure, and a SiO_2_ coating by sputtering.

Microfluidic channels were produced subsequently after IDT patterning on the wafer scale using a custom technique for dry film resist (DFR) lamination and photolithography,^39^ defining first the channel walls and second the channel cover with high geometrical and placement accuracy. The patterning was realized by a two-step lamination process, including a first DFR (DF3550, Nagase ChemteX) lamination and laser-based photolithographic patterning of channel side walls (height: 50 μm, thickness: 50 μm), followed by a second lamination and likewise patterning of the cover layer (thickness 50 μm). As for the pIDTs, all steps for channel patterning were realized by rapid-prototyping laser photolithography. The resulting microfluidic channel on each chip had dimensions of 150 μm inner width and 50 μm inner height.

The acoustofluidic chips were mounted in a custom chip holder, allowing optical access through the chip along the surface-normal axis by an inverted microscope configuration and a fluidic connection to the pump via PEEK tubing and fluid interconnection blocks pressed on the chip surface and sealed via PDMS foil. Electrical radiofrequency-suited connection was realized via custom PCBs with gold-coated spring pins, conductor-backed coplanar waveguides (CBCPW) of 50 Ω characteristic impedance, and SMA connectors for the cabling. A pressure pump unit (LineUp Flow EZ and Flow Unit M, Fluigent) provided a speed-regulated flow. This flow was connected to the chip via PTFE tubing with a 1/32” inner diameter (Figure 1c). Only one fluid inlet was used; i.e., the cells were not focused by a sheath flow.

### Estimation of Object Velocities

For bead measurements, the pump was set to 0.5 μL/min, which theoretically results in a 1.1 μm/ms average particle speed and a maximum of 2.2 μm/ms in the middle of the flow. In the camera recording, particles were flowing with a speed of 10 to 20 pixels/frame or 2.2 to 4.3 μm/ms (20× objective lens, 6.5 μm pixel size). The optoacoustic data show an average of 1.3 ms of interrogation time fwhm for 3 μm diameter beads, which results in a 2.3 μm/ms flow speed.

### Optoacoustic and Camera Setup

For optical excitation of cells and beads in flow (Figure 1c), a dye laser (Credo Dye N, Sirah Lasertechnik GmbH) utilizing Rhodamine 6G tuned for 570 nm emission was used. The dye laser was pumped by a <100 kHz, 5 ns 532 nm diode-pumped solid-state laser (IS80–2-L, EdgeWave GmbH). Laser pulses were triggered using a function generator (DG1022A, Rigol) at a pulse-repetition rate of 10 kHz and a focus spot of approximately 1 μm diameter inside the microfluidic channel. A rotatable half-wave plate (HWP, AHWP05M–600, Thorlabs) and a polarizing beam splitter (CCM1-PBS251/M, Thorlabs) control the laser power. For further fractional reduction of the laser power, a filter wheel with neutral density filters was used (FW2AND, Thorlabs). To trigger the acquisition of pIDT signals synchronized with the laser pulses, a fraction of the beam (quartz coverslip) was recorded with a high-speed photodiode (818-BB-20, Newport) and used to trigger acquisition. The excitation light was coupled by a 50:50 beam splitter (CCM1-BS013/M, Thorlabs) into the infinity space of a microscope consisting of a long working distance objective (20× Plan Apochromat 0.42 NA, Mitutoyo) and tube lens (AC254–200-A-ML, Thorlabs). For parallel imaging of the cells in the microfluidic channel, a CMOS camera with frame rates up to 1 kHz (Prime, Teledyne Photometrics, ROI size (31 × 31 μm) and exposure time (1 ms) limited the effective frame rate to 670 fps) and protected from the laser light by a short-pass filter (FES0500, Thorlabs) was used. Light was provided by white light top illumination. Since the chip substrate is birefringent, a polarizing beam splitter (PBS251, Thorlabs) and rotatable HWP (AHWP10M–600, Thorlabs) were added to control the polarization, suppressing double images. The chip was mounted on an XY-stage (2× MT1/M, Thorlabs) with pulsed laser illumination and imaging from the bottom side. For focusing, the objective lens was mounted on a *z*-axis translation mount (SM1ZA, Thorlabs).

Electrical signals from the pIDT (Figure 2a) were amplified by 60 dB using a variable gain amplifier (DHPVA-100, Femto) and digitized by a two-channel 1 GS/s/channel PCIe card (GS161G2, GaGe). Cell detection events were identified after selecting the time-of-flight region for the optical focus in the microfluidic channel and using the power spectrum density as a signal measure (Figure S1). The choice of time-of-flight selection was independently controlled by measuring the SNR for multiple possible selection windows (Figure 2b). Simultaneously, objects flowing through the channel in the focal area were recorded by a CMOS camera recording at 670 frames per second (fps) and subsequently analyzed by synchronization of the camera detection with the OA signal measured by the pIDT to identify coregistered events (Figure S1). To confirm the operation frequencies of the chips and estimate the travel time of surface acoustic waves in the respective layout, we generated test pIDT signals by aiming the laser beam at an aluminum test target on the chip’s surface next to the channel.

### Mammalian and Bacterial Cell Culture

Red blood cells (RBCs) were isolated by Percoll density gradient centrifugation following the manufacturer’s instructions (Sigma-Aldrich, St. Louis, Missouri, USA). Briefly, sheep blood (Thermo Scientific, Massachusetts, USA) was diluted with PBS and layered onto a Percoll working solution with a density of 1.07 g/mL and centrifuged at 10 000 *g* for 20 min at 4 °C. The isolated RBC layer was carefully collected and subsequently washed with PBS.

B16-F10 cells were obtained from the American Type Culture Collection (ATCC) and cultured according to the supplier’s recommendations. In short, cells were maintained in Dulbecco’s modified Eagle’s medium (DMEM) supplemented with 10% fetal bovine serum (FBS) and 1% penicillin–streptomycin. In this study, early passage cells (P4-6) were collected to ensure pigmentation.

### Sample Preparation and Measurement

Samples were filtered through a 40 μm cell strainer (352340, Falcon) to remove aggregates. The total dilution was chosen to achieve 10–20 detected particles per second.

For data acquisition, camera recording and digitizer acquisition were started simultaneously. For efficient recording, custom processing, and storage, a custom C++ code was used. Despite synchronization, the different control of the camera and acquisition results in an offset that was compensated for in the analysis (see below).

### Data Analysis

All analyses were performed using MATLAB 2023b. Digitized signals from the pIDT were time-gated, i.e., selectively cropped to isolate the temporal region associated with the OA signal emanating from the cells (Figure S1) and subsequently Fourier transformed. Considering the electrical bandwidth of the pIDTs, the overall OA signal for the pulse was considered the maximum amplitude of the power spectral density around ±10% of the center frequency of the pIDT (i.e., 10, 25, 50, 100 MHz). These curves over time (i.e., per laser pulse) were baseline corrected by subtracting the sliding mean with a window size of 10 000. Afterward, this OA signal was averaged with a Gaussian filter with a window length of 15 and a standard deviation of 2.3 to reduce noise, eventually yielding the final “OA signal” used for cell detection. To calculate the SNR of the OA signal, we divided the OA signal of coregistered events by the noise (standard deviation of ∼1000 trigger events with no visible particle transition). The ROIs of the camera images underwent baseline correction using a sliding mean calculation with a window size of 1000, applied to each pixel across the time series. Following this, the absolute value was computed for each pixel. This approach allowed for the registration of both brighter and darker areas, as some particles refract light and create a bright spot adjacent to the particle. In such cases, a simple averaging of the grayscale value would not detect any changes, as the bright area cancels out the dark area, leading to missed particle detections. These values are defined as the “camera signal” shown in the figures. For coregistration of events in the OA and camera time trace, peaks were identified with a threshold of 5× the standard deviation of noise. To evaluate this threshold, the receiver operating characteristics (ROC) were calculated and are shown exemplarily for the 50 MHz, 3 mm focal length pIDT (Figure S3). Here, we varied the threshold to detect peaks from 1σ to 40σ (SD of the noise). The true positive rate was calculated as TPR = *N*_coregistered_/*N*_camera_ and the false negative rate as FPR = *N*_only OA_/(*N*_frames,total_ – *N*_camera_). Here, *N*_camera_ is the total number of events detected by the camera, *N*_coregistered_ is the number of events successfully coregistered with the pIDT, *N*_only OA_ is the number of events detected only by the OA system but not the camera, and *N*_frames,total_ is the total number of camera frames. The area under the ROC curve (AUC) provides an overall measure of the detection performance. The AUC quantifies the overall performance of the detector, where a value of 1 indicates perfect discrimination, and 0.5 represents random guessing. The ROC curve also illustrates the trade-off between sensitivity (TPR) and specificity (1-FPR) at various thresholds. Lower thresholds improve sensitivity, capturing more true positives, but may increase false positives, while higher thresholds reduce false positives at the cost of missed detections. The chosen 5σ threshold reflects a practical balance, achieving a high true positive rate while keeping false positives low, as supported by its consistent performance across different particle types. To compensate for a variable offset between the start of the digitalization of the pIDT signals and the recording of the camera (∼10–20 ms), the positions of the 50 highest peaks were matched against each other for the smallest offset against a peak in the respective other time series. The median offset was considered the real technical offset and was corrected by shifting the delayed time series. Subsequently, events registered in both time series were identified by finding peaks that were closer than 5 ms to each other.

## Supporting Information Available

Details on data analysis; determination of data analysis thresholds; frequency characteristics of the electrical reflection coefficient; trace for 3 μm polystyrene beads; Figure as in 3c including the signal; correlation plots of OA and camera signals; tabular overview of all recorded data (PDF)
