# Supplementary material for: Chip-Based Optoacoustic Single-Cell Detection in Flow Using Point-Source Optimized Surface Acoustic Wave Transducers
Source: ACS Appl Mater Interfaces. 2025 Feb 25;17(10):14903–11. doi: 10.1021/acsami.4c20182 (PMC11912191; doi:10.1021/acsami.4c20182)
Supplement: Supplementary file 1 — am4c20182_si_001.pdf [file am4c20182_si_001.pdf]

Chip-based optoacoustic single cell detection in flow using point-source optimized surface acoustic wave transducers.

Simon Göllner[1,2], Melanie Colditz[3], Yishu Huang[1,2], Hagen Schmidt[3*], Andreas Winkler[3*] and Andre C. Stiel[1,2,4*]

1: Institute of Biological and Medical Imaging, Helmholtz Zentrum München, 85764 Neuherberg, Bavaria, Germany.
2: Chair of Biological Imaging, Central Institute for Translational Cancer Research (TranslaTUM), School of Medicine and Health & School of Computation, Information and Technology, Technical University of Munich, Munich, Germany
3: Leibniz Institute for Solid State and Materials Research, SAWLab Saxony, 01069 Dresden, Saxony, Germany
4: Protein Engineering for Superresolution Microscopy Lab, University of Regensburg, 93053 Regensburg, Bavaria, Germany

* Correspondence to: andre.stiel@helmholtz-muenchen.de, h.schmidt@ifw-dresden.de or a.winkler@ifw-dresden.de

Supporting Information

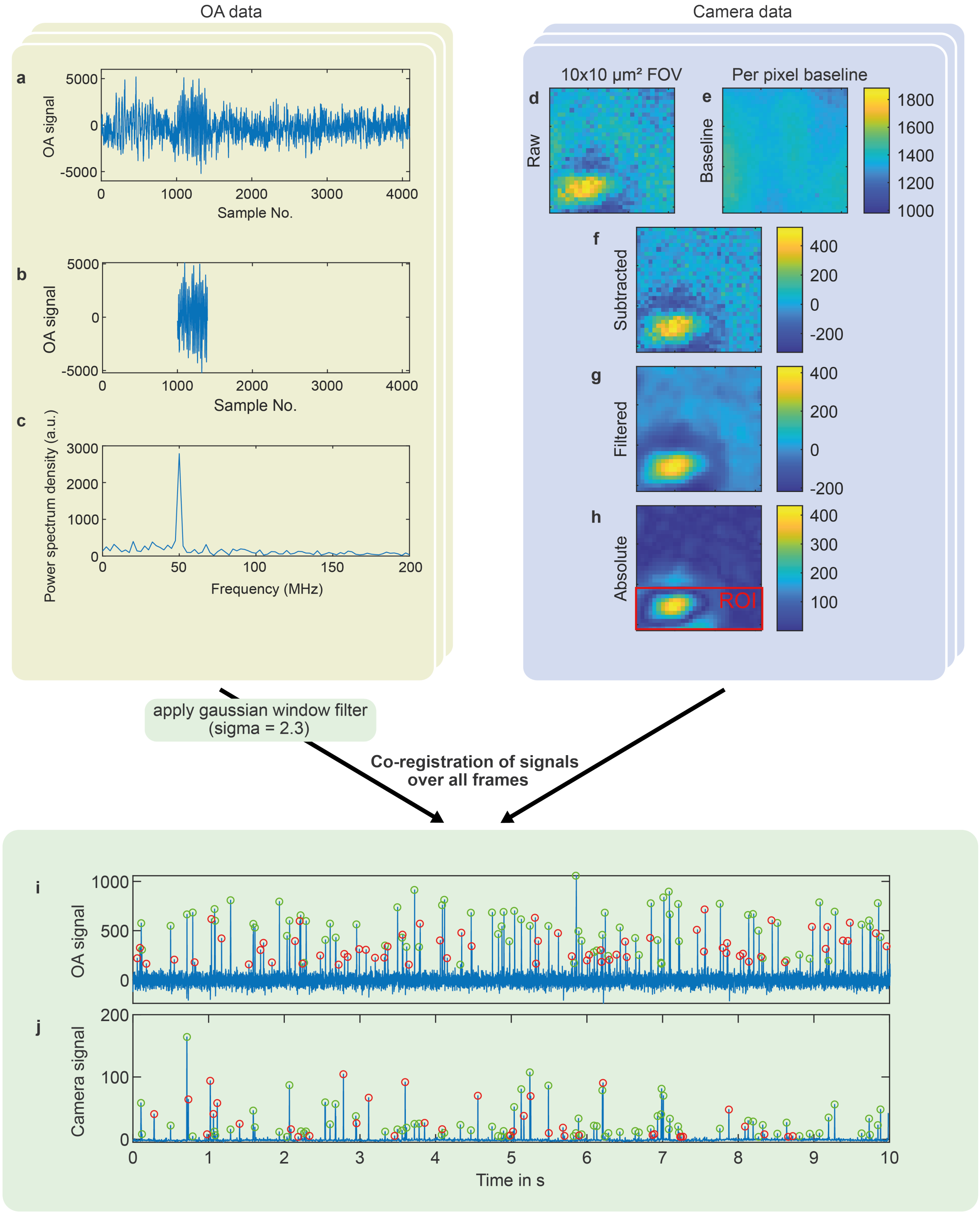

Figure S1: Schematic illustration of the signal processing workflow demonstrated using an RBC measurement with a 50 MHz pIDT. (**a**) Raw OA signal acquired by pIDT. (**b**) The pIDT signal is trimmed based on the time-of-flight corresponding to the OA signal that originates from the sample particle. (**c**) The power spectrum shows a peak at the pIDT center frequency (50 MHz). (**d**) 10x10 µm² frame recorded at 670 fps using the custom microscope focusing into the microfluidic channel. An RBC is visible in the bottom left of the frame. (**e**) Each pixel's baseline is calculated using a moving average over 100 frames to compensate changes in the image over time. (**f**) The baseline is subtracted from the raw frame. (**g**) A gaussian filter (σ = 1) is applied to remove noise. (**h**) Negative pixel values are inverted to avoid situations where negative values average out positive ones, which results in some cases in undetected particles.

The red rectangle indicates the ROI which is averaged for particle detection. (**i**) Processed OA signal over time. The OA signal refers to the amplitude at the center frequency of the pIDT (see above a-c); events are augmented by using a gaussian window filter (σ=2.3). Co-registered peaks are highlighted with green circles, all others are highlighted in red. (**j**) Processed camera signal over time. The camera signal refers to the average of the ROI from Suppl. Figure 2h and is filtered with a median filter to remove spikes in the camera signal. Co-registered peaks are highlighted with green circles, all others are highlighted in red.

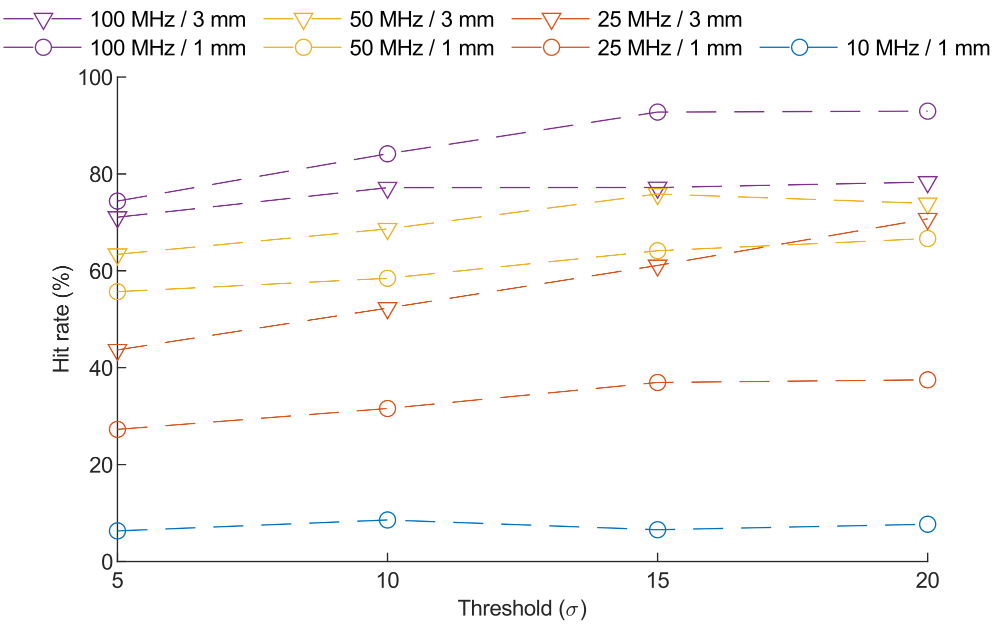

Figure S2: Hit rate in respect to the camera signal threshold for all pIDTs measuring beads. The camera threshold is given in multiples of the standard deviation of noise (σ). An increase of the threshold results overall in an increase of the hit rate, but reduction in the number of detected particles.

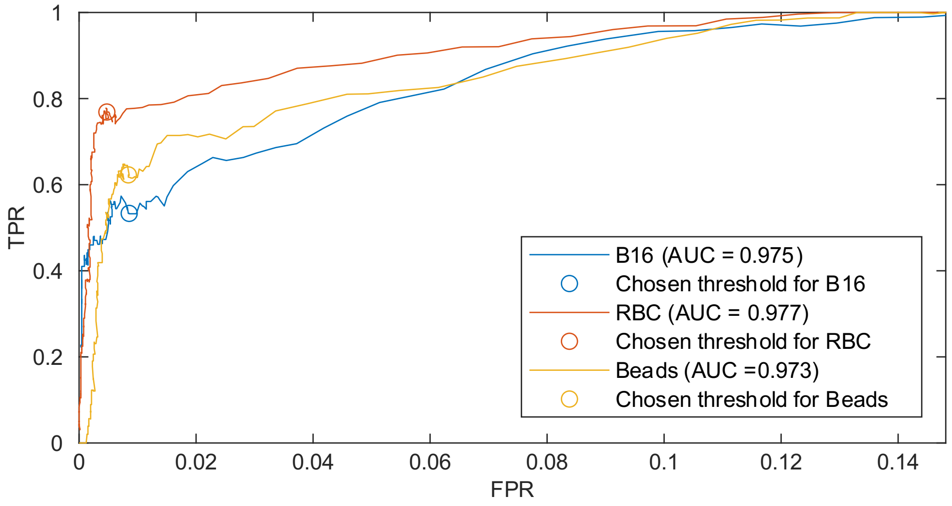

Figure S3: Receiver operating characteristic (ROC) curves exemplarily shown for measurements using the 50 MHz pIDT with 3 mm focal length. The chosen threshold of 5σ shows a good balance between high true positive rate (TPR), while keeping the false positive rate (FPR) to a minimum.

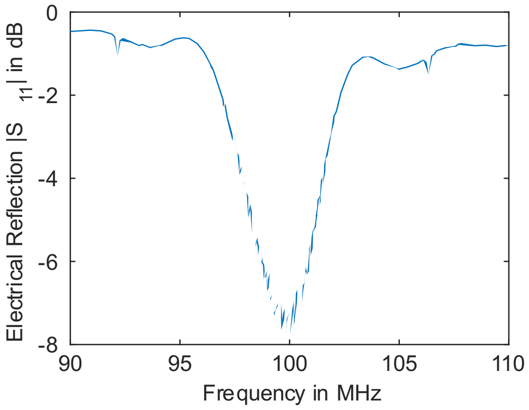

Figure S4: Frequency characteristics of electrical reflection coefficient $|S_{11}|$. Shown is exemplary the coefficient for an 100MHz pIDT with 1mm focal length

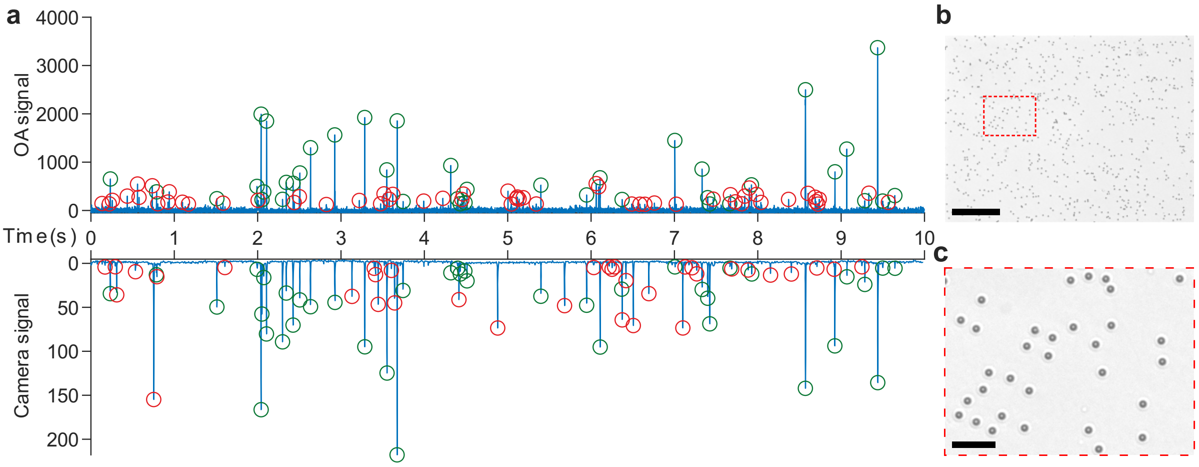

Figure S5: Exemplary traces of 3 µm polystyrene beads. (**a**) Trace of 3 µm beads. This measurement was done using the 100 MHz pIDT with 3 mm focal length. 48 events were co-registered out of 65 events detected by the camera (74% hit rate). (**b**) Microscope image of an aliquot of 3 µm beads (scale bar 125 µm). (**c**) Section of (**b**) (scale bar 25 µm).

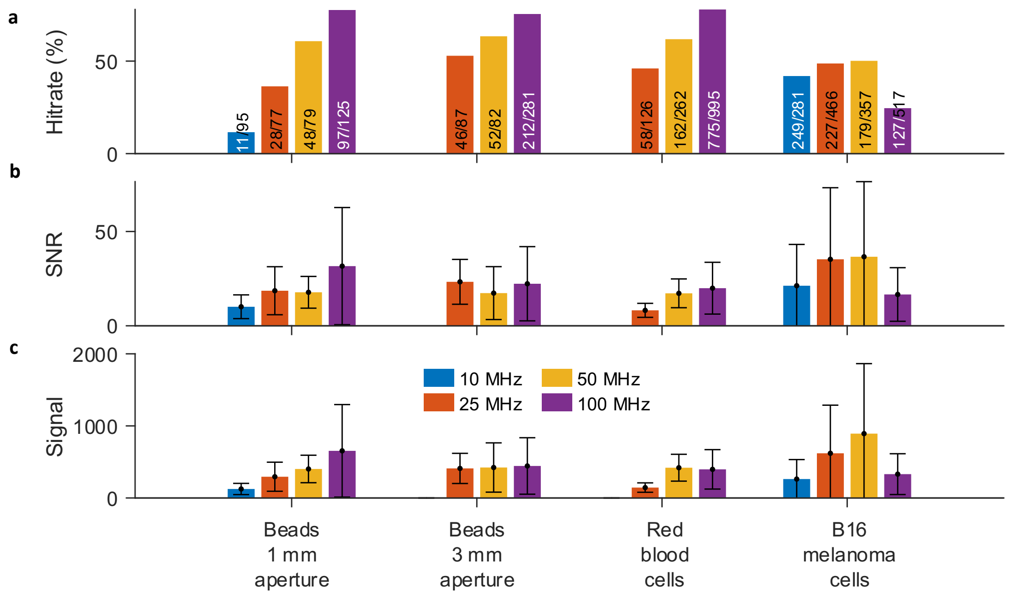

Figure S6: Characteristics of the measurements regarding OA signal, SNR and hit rate. In the (**b**) and (**c**) bars depict the mean, the error bars depict the standard deviation. Due to the dimension of the pIDT, there is no 10 MHz chip with a 3 mm focal length. Red blood cells could not be detected using the 10 MHz pIDT.

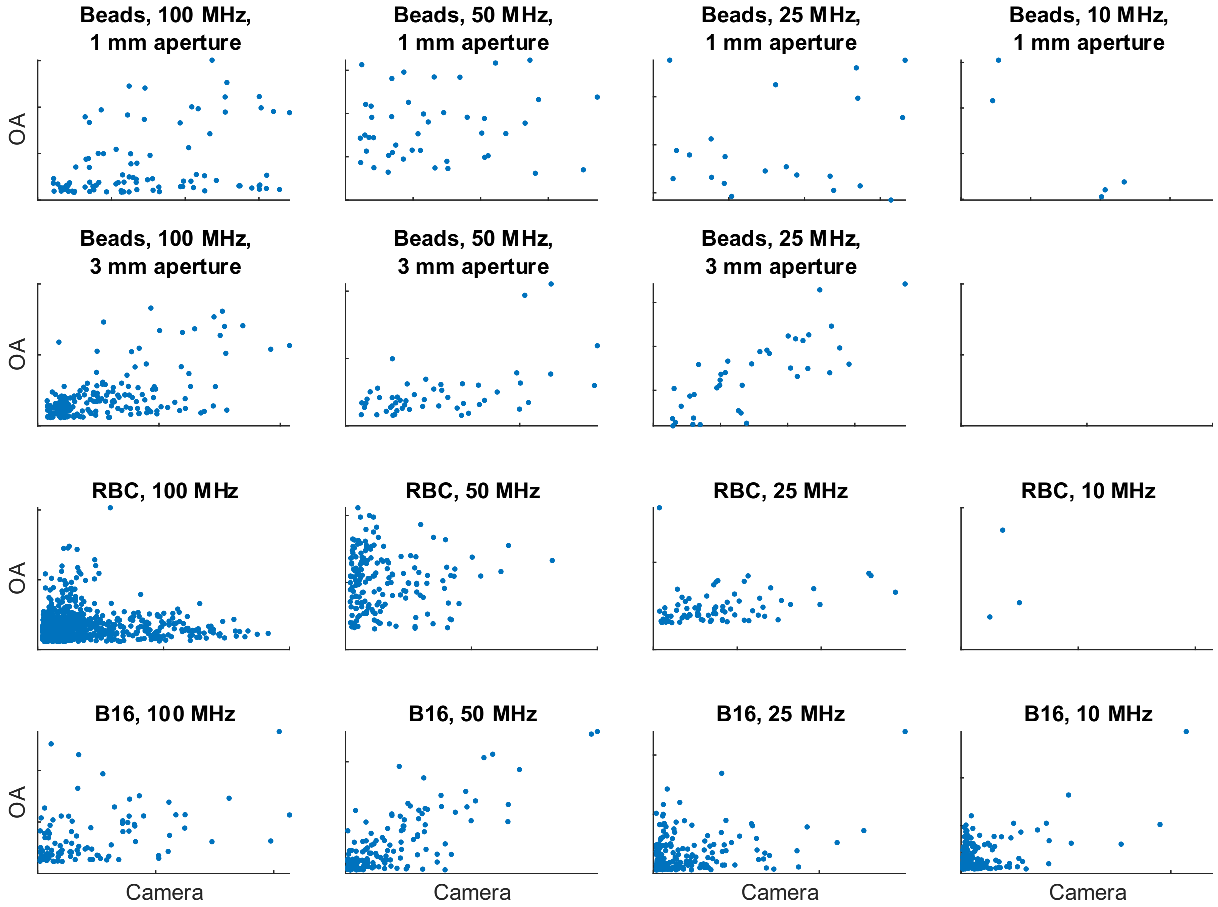

Figure S7: Correlation of camera and OA signal for co-registered events. First row shows beads measurements with the 1 mm focal length pIDTs, second row beads measurements with the 3 mm focal length pIDTs, third row red blood cells, fourth row B16 cells. Each column shows the measurements of a single pIDT (100, 50, 25, 10 MHz).

					Co-registered						Detected only by OA			Detected only by camera		
	Freq. in MHz	Focal length in mm	Noise OA	Noise Camera	No. of co-registered events	Hit rate (No. Co-registered divided by all events detected by camera)	Average camera signal	Std. dev. of camera signal	Average OA signal	Std. dev. of OA signal	No. of events	Average OA signal	Std. dev. of OA signal	No. of events	Average camera signal	Std. dev. of camera signal
**Beads**	100	1	16	0,8	93	74	29	18	368	357	20	110	33	32	12	10
	50	1	23	0,7	44	56	26	19	335	153	40	262	110	35	22	20
	25	1	30	0,8	21	27	33	21	390	193	21	318	238	56	19	18
	10	1	9	0,8	6	6	37	24	99	65	9	64	20	89	27	22
	100	3	23	0,8	199	71	26	21	395	319	71	217	119	81	18	17
	50	3	31	0,7	52	63	26	18	482	376	33	283	157	30	16	13
	25	3	12	0,7	38	44	29	18	118	41	11	86	29	49	12	7
**RBC**	100	3	23	0,8	763	77	35	34	348	209	157	215	121	227	15	16
	50	3	31	0,7	175	66	29	28	517	208	142	327	138	89	32	32
	25	3	12	0,7	75	59	38	30	98	40	12	83	19	52	12	12
	10	1	9	0,8	4	2	16	18	187	163	13	111	62	167	13	11
**B16**	100	3	23	0,8	107	21	85	92	338	244	66	256	202	403	21	37
	50	3	31	0,7	167	46	67	77	1154	1237	253	342	320	196	18	21
	25	3	12	0,7	201	43	37	47	318	316	535	188	184	266	18	23
	10	1	9	0,8	210	35	38	69	170	160	532	106	71	383	20	24

Table S1: Overview on all experimental conditions and obtained results. The color coding is for each column and sample individually done. Color coding per column from red (highest) to blue (lowest).
